# Long-term symptoms in dizzy patients examined in a university clinic

**DOI:** 10.1186/1472-6815-9-2

**Published:** 2009-05-16

**Authors:** Kjersti Wilhelmsen, Anne Elisabeth Ljunggren, Frederik Goplen, Geir Egil Eide, Stein Helge G Nordahl

**Affiliations:** 1 Department of Public Health and Primary Health Care, Section for Physiotherapy Science, University of Bergen, Bergen, Norway; 2 National Centre for Vestibular Disorders, Department of Otorhinolaryngology/Head and Neck Surgery, Haukeland University Hospital, Bergen, Norway; 3 Department of Physiotherapy, Bergen University College, Bergen, Norway; 4 Department of Surgical Sciences, University of Bergen, Bergen, Norway; 5 Centre for Clinical Research, Haukeland University Hospital, Bergen, Norway; 6 Department of Public Health and Primary Health Care, Research Group on Lifestyle Epidemiology, University of Bergen, Bergen, Norway

## Abstract

**Background:**

The long-term course of dizziness was investigated combining medical chart and survey data. The survey was undertaken median (interquartile range (IQR)) 4.6 (4.3) years after the initial medical examination.

**Methods:**

Chart data comprised sex, age, diagnosis, symptom duration, postural sway and neck pain. Survey data comprised symptom severity assessed by the Vertigo Symptom Scale – Short Form (VSS-SF), and data regarding current state of dizziness, medication, neck pain and other chronic conditions.

**Results:**

The sample consisted of 503 patients, the mean (standard deviation (SD)) age was 50.0 (11.6) years, women being slightly overrepresented (60%). Severe problems with dizziness (VSS-SF mean (SD) 13.9, (10.8)) were indicated in the total group and in 5 of 6 diagnostic sub-groups. Vertigo/balance- and autonomic/anxiety-related symptoms were present in all groups. Current dizziness was confirmed by 73% who had significantly more severe problems than the non-dizzy (VSS-SF mean (SD): 17.2 (10.1) versus 5.0 (7.3)). Symptoms were related to vertigo/balance more than to autonomic/anxiety (test of interaction p < 0.001).

Based on simple logistic regression analysis, sex, symptom duration, neck pain, sway and diagnoses predicted dizziness. Symptom duration and neck pain remained predictors in the adjusted analysis. Age, symptom duration, neck pain, sway and diagnoses predicted vertigo/balance-related dizziness in both regression analyses. Sex, neck pain and sway predicted development of autonomic/anxiety-related dizziness according to simple regression analysis, while only neck pain remained a significant predictor in the adjusted analysis. With respect to diagnosis, simple regression analysis showed significant reduced likelihood for development of dizziness in all vestibular sub-groups when compared to the non-otogenic dizziness group. With respect to vertigo/balance- and autonomic/anxiety-related symptoms, the implication of diagnostic belonging varied. No effect of diagnoses was seen in adjusted analyses.

**Conclusion:**

The majority of patients had persistent and severe problems with dizziness. The wait-and-see attitude before referral to specialist institutions may be questioned. Early, active movements seem necessary, and attention should be paid to the presence of neck pain. Diagnoses had limited prognostic value. Questionnaire-based evaluations could assist in classification and identification of type of dizziness and thereby provide a better basis for specific rehabilitation.

## Background

For many patients experiencing vertigo and dizziness, symptoms resolve spontaneously within a short period of time [1]. According to Kroenke and co-workers, symptoms resolved within 2 weeks in almost 30% [2], and among those with remaining symptoms, 50% indicated symptom resolution at the one year follow-up [1]. However, for some, dizziness persists and lack of improvement has been reported in 11% [3]. To cope in every-day life situations, movements and activities may be restricted and even avoided, which over time may result in functional problems and also secondary symptoms in the musculoskeletal system [4]. Fear of provoking symptoms may lead the patient into a vicious circle with physical and psychosocial consequences influencing health-related quality of life.

Referral rates from primary care to specialist units vary. The number of visits, length of history [5] as well as functional impact of dizziness [6], guides referral. A study from the United States reported a referral rate of 9% [7]. In Scotland 4% was reported [8], while referral rates from England vary from 8–20% [3,5,6,9]. The policy of wait-and-see represents a challenge in later diagnostic processes as characteristic symptoms and signs of the acute stage are lacking. Patients may have problems recalling past events, and the description of problems is vague and non-specific [5]. In about 1 of 7 cases the cause for dizziness is not established [10], while 44% [10] to 65% [11] of cases are diagnosed with peripheral vestibular aetiology. Relatively little seems to be known regarding symptoms and signs in a long-term course.

The purpose of the present study was to explore the long-term course of dizziness in some patients referred to a balance clinic in a university hospital with complaints of persistent dizziness. It was also an aim to explore the predictive ability of some factors identified in the medical examination.

## Methods

Patients between 18 and 70 years with the following diagnoses in the vestibular category were included in the present study: Menière's disease, vestibular schwannomas, benign paroxysmal positional vertigo and vestibular neuritis. Patients with central vestibular disorders were excluded. Patients with non-vestibular dizziness were assigned to two groups. Those with significant neck symptoms accompanying dizziness were diagnosed as "cervical dizziness" when no other explanation was found. The remaining patients diagnosed in the non-vestibular group were classified as "non-otogenic" when no other cause was found. The inclusion criteria were met by 821 patients.

All included patients had been examined for suspected vestibular disorders. Patients were referred from general practice and other specialist care units. The extent of the medical examination varied accordingly, but most of the referred patients had undergone evaluation by an otolaryngologist. The evaluation in our clinic included audiometry (pure tone and speech) and clinical ear, nose and throat examination. The clinical examination was associated with laboratory investigations comprising static posturography, electronystagmography with bithermal caloric tests, testing for spontaneous and positional nystagmus, ocular smooth pursuit and saccades. The final diagnosis was set retrospectively by an experienced otolaryngologist according to medical chart information.

The study combined existing medical chart data (1992–2001), and postal survey data collected in spring 2002. The median (interquartile range (IQR)) time period from the patients' first medical examination to the survey, was 4.6 (4.3) years.

Medical chart data were sex, age, neck pain (yes/no) and balance in the standing position (registered path length in mm of centre of pressure during quiet stance for one minute, eyes closed) by static stabilometry (Cosmogamma^©^, Bologna Italy) [12]. Symptom duration from debut to the medical examination was calculated.

Survey data were age, use of vestibular sedatives, presence of neck pain and of other chronic conditions. Apart from age, the questions were coded as yes/no. Current state of dizziness was identified by the following question: "Are you still bothered with dizziness?" with response categories: Yes/No. Recent spells of dizziness were identified by "When did you have your last episode of dizziness?" with response categories: 1) During the day, 2) Within last week, 3) Within last month, 4) More than a month ago. Categories 1–3 were collapsed to indicate "recent episode", i.e. within the last month. Symptom duration from debut to survey, and time interval between medical examination and survey, were calculated.

Severity of symptoms was assessed in the survey by a Norwegian version of the Vertigo Symptom Scale – Short Form (VSS-SF) [13] developed by Yardley et al. [14]. The psychometric properties of the translated version was explored and found satisfactory [13]. The scale consists of 15 items and has two sub-scales: vertigo/balance- (VSS-V, 8 items) and autonomic/anxiety- (VSS-A, 7 items) related symptoms. Frequency of symptoms during the past month is marked on a 5-point Likert scale ranging from "never" (0) to "very often, almost daily" (4). VSS-SF total score ranges 0–60 points, VSS-V ranges 0–32 and VSS-A ranges 0–28 points. Severe dizziness is indicated by a score ≥ 12 points on the total scale [15]. Cut-points indicating no dizziness/dizziness have been identified for the scale and sub-scales: VSS-SF ≤ 6.5, VSS-V ≤ 2.5 and VSS-A ≤ 3.5 [13].

The study was approved by the Regional Committee for Medical Research Ethics in Western Norway as part of a larger study. Written informed consent was obtained from all patients.

### Statistical analyses

Demographic and survey data were compared between the diagnostic groups, and reported as mean, median, standard deviation (SD), interquartile range (IQR) and 95% confidence interval (CI). Distribution of symptom scores was examined by q-q plots and by comparing mean and median scores of the VSS-SF and sub-scales. As normality could be assumed, parametric statistical methodology was used. Mean sub-scale score was obtained by dividing the sub-scale sum score with the corresponding number of items. One-way analysis of variance (ANOVA) with Bonferroni's post-hoc tests was used to explore differences between groups in normal continuous data, for non-normal continuous data the Kruskal-Wallis test was used. Pearson's chi-square statistical methodology was used to explore differences between groups in dichotomous data. Statistical significance was set at p ≤ 0.05. SPSS version 15 for Windows was used for all analyses.

One-way ANOVA with Bonferroni's post-hoc test was performed to determine the significant differences between diagnostic group means. In order to analyse possible dependency of the VSS-SF on the state of dizziness and diagnosis, a two-way ANOVA was performed with dizzy/not dizzy (2 categories) and diagnostic groups (6 categories) as grouping factors. This allowed for testing if symptom score differed in dizzy and not dizzy, and if the difference varied across diagnostic groups (interaction). To analyse the possible dependency of the VSS sub-scales on the state of dizziness and diagnosis, ANOVA was performed with dizzy/not dizzy (2 categories) and diagnostic group (6 categories) as grouping factors, and sub-scale as repeated factor (2 sub-scales). This allowed for testing complex interactions, e.g. if difference in sub-scale scores between dizzy and not dizzy was the same for both sub-scales, and if it was modified by diagnostic group. The general linear model (GLM) procedure with the repeated measures option of SPSS was applied [16].

Simple (unadjusted) and multiple (adjusted) logistic regression analyses were used to identify predictors of overall dizziness *and *type of dizziness. Dependent variables were VSS-SF, VSS-V and VSS-A using the respective cut-off values indicating the absence or presence of symptoms. Sex, age (in 10 year intervals), neck pain, path length (converted to meters), symptom duration at medical examination (short-term ≤ 6 months; long-term > 6 months) and diagnosis (reference category: non-otogenic dizziness, *n *= 130) retrieved from the medical charts were used as explanatory variables. The complete model is presented in the results.

## Results

Of 2067 patients, 821 (40%) met the inclusion criteria and 549 (67%) patients returned the questionnaire. Closer inspection showed a varying number of missing items in 86 of the returned VSS-SF forms. Imputation was done in 40 forms, i.e. forms lacking one or two items on the respective sub-scales, 46 forms were discarded. The final sample therefore consisted of 503 responders with sufficient data for calculation of symptom scores on the VSS-SF. In the group, 385 patients responded to the first mailing (early responders), while 118 patients responded to posted reminders (late responders). The remaining 272 patients did not return the questionnaire (non-responders).

At the time of the medical examination the median (IQR) symptom duration was 1.8 (4.0) years. The median (IQR) path length measured by the balance platform was 829 (668) mm. Neck pain was reported by 27%. Details of demographic information from medical charts according to diagnostic groups are presented in Table 1.

**Table 1:** Demographic characteristic

**Diagnostic groups**	**n (%)^a^**	**Symptom duration^b^** **median (IQR)**	**Path length^c^** **median (IQR)**	**Neck pain^d^** **n (%)**
Menière's disease	92 (18)	26.5 (65)	739 (527)	16 (17)
Vestibular schwannoma	40 (8)	33.0 (32)	1007 (867)	2 (5)
Benign positional vertigo	90 (18)	18.0 (40)	809 (551)	28 (31)
Vestibular neuritis	89 (18)	8.0 (24)	822 (644)	19 (21)
Non-otogenic dizziness	130 (26)	24.5 (54)	941 (765)	37 (29)
Cervical dizziness	62 (12)	32.5 (56)	777 (649)	33 (53)
Total N	503 (100)	442 (100)	467 (668)	503 (100)
P value		≤ 0.01^e^	0.03^e^	≤ 0.01^f^

Demographic characteristic at the medical examination according to diagnostic groups in N = 503 patients with dizziness examined in the period 1992–2001 in Bergen, Norway

IQR: interquartile range

^a ^Distribution of patients within each diagnostic group in relation to total group

^b ^Symptom duration in months

^c ^Path length in mm, eyes closed condition at the medical examination

^d ^Neck pain at the medical examination

^e ^P values according to the Kruskal-Wallis test

^f ^P values according to Pearson's chi-square test

At the time of the survey, the mean (SD) age was 50.0 (11.6) years. Patients with vestibular schwannoma were significantly older than patients in the non-otogenic and cervicogenic groups (p < 0.001). Women were slightly overrepresented (60%), but no difference in sex distribution was seen between the diagnostic groups (p = 0.33). Only 8% used vestibular sedatives, 59% (n = 298) indicated neck pain, and 61% (n = 299) reported other chronic conditions. The median symptom duration (IQR) was 7.1 (5.5) years. In the total sample, the mean (SD) symptom score on the VSS-SF was 13.9 (10.8); on the VSS-V it was 7.5 (6.8), and on the VSS-A it was 6.4 (5.3). Significant higher scores were seen in the non-otogenic compared to the vestibular schwannoma group on the total and sub-scale scores. Details of scores in the diagnostic groups are presented in Table 2. Dizziness was reported by 73% of the patients, and out of these, 80% confirmed dizzy spells within the last month. The mean total and sub-scale scores in diagnostic groups according to current state of dizziness are given in Table 3. A significant association (p < 0.001) was found between dizziness and neck pain. Dizziness was not associated with other chronic conditions (p = 0.82).

**Table 2:** Symptom scores in total sample

**Diagnostic groups**	**VSS-SF** **mean (SD)**	**VSS-V** **mean (SD)**	**VSS-A** **mean (SD)**
Menière's disease	12.89 (10.70)	6.91 (6.36)	5.98 (5.21)
Vestibular schwannoma	8.65 (7.71)	4.35 (4.66)	4.30 (4.12)
Benign positional vertigo	13.80 (11.38)	7.92 (7.32)	5.88 (5.47)
Vestibular neuritis	13.43 (11.28)	7.15 (7.00)	6.28 (5.26)
Non-otogenic dizziness	16.24 (10.90)	8.72 (6.98)	7.52 (5.49)
Cervical dizziness	14.84 (9.98)	7.94 (6.55)	6.90 (4.85)
Total group	13.92 (10.82)	7.52 (6.79)	6.39 (5.28)

The Vertigo Symptom Scale Short Form (VSS-SF), the vertigo/balance sub-scale (VSS-V) and autonomic/anxiety sub-scale (VSS-A) according to diagnostic groups in N = 494 patients examined in the period 1992–2001 in Bergen, Norway

SD: Standard deviation

**Table 3:** Distribution of patients and symptom scores according to diagnosis

**Diagnostic groups**	**N (%)^d^**	**VSS-SF** **mean (SD)**	**VSS-V** **standardized mean (SD)**	**VSS-A** **standardized mean (SD)**
*Dizzy*				
Menière's disease	64 (72)	15.95 (10.28)	1.13 (0.78)	0.99 (0.75)
Vestibular schwannoma	23 (61)	13.00 (7.11)	0.84 (0.59)	0.90 (0.59)
Benign positional vertigo	61 (69)	17.49 (10.19)	1.29 (0.83)	1.03 (0.79)
Vestibular neuritis	60 (68)	18.12 (10.43)	1.25 (0.83)	1.15 (0.73)
Non-otogenic dizziness	111 (86)	17.87 (10.49)	1.23 (0.84)	1.15 (0.77)
Cervical dizziness	46 (74)	17.65 (9.58)	1.24 (0.80)	1.11 (0.70)
Total dizzy sub-group	365 (74)	17.18 (10.13)	1.20 (0.81)	1.08 (0.74)
				
*Not dizzy*				
Menière's disease	25 (28)	5.52 (8.13)	0.23 (0.45)	0.53 (0.71)
Vestibular schwannoma	15 (40)	2.47 (3.46)	0.11 (0.19)	0.23 (0.32)
Benign positional vertigo	27 (31)	5.89 (9.94)	0.35 (0.75)	0.44 (0.61)
Vestibular neuritis	28 (32)	3.50 (5.07)	0.15 (0.30)	0.33 (0.44)
Non-otogenic dizziness	18 (14)	5.83 (7.63)	0.21 (0.51)	0.59 (0.73)
Cervical dizziness	16 (26)	6.75 (5.95)	0.29 (0.33)	0.63 (0.55)
Total not dizzy sub-group	129 (26)	5.00 (7.28)	0.23 (0.48)	0.46 (0.59)

Number of participants and distribution according to diagnostic category and scores on the Vertigo Symptom Scale Short Form (VSS-SF), the vertigo/balance (VSS-V^a^) and the autonomic/anxiety (VSS-A^b^) sub-scales according to dizziness and diagnostic group^c ^in N = 494 patients examined in the period 1992–2001 in Bergen, Norway

^a ^VSS-V sum score divided by number of scale items (8)

^b ^VSS-A sum score divided by number of scale items (7)

^c ^Analyses of variance showed significant differences between dizzy and not dizzy patients for all scales (VSS-SF, VSS-V, VSS-A). The difference was significantly larger for VSS-V than for VSS-A. Diagnostic group had no significant effect on any of the scales

SD: Standard deviation

The results from the two- and three-way ANOVA showed that diagnosis could be eliminated as it had no significant effect on the severity of symptoms. As expected, the mean symptom score on the VSS-SF differed significantly (p < 0.01) between dizzy and not dizzy patients; the mean difference was estimated as a score of 12.18 (95% CI: 10.27, 14.08). On the sub-scales, the mean difference in symptom scores between dizzy and not dizzy was larger for the VSS-V than for the VSS-A (test of interaction p < 0.001) estimated as 0.98 (95% CI: 0.83, 1.12) on the VSS-V, and as 0.63 (95% CI: 0.48, 0.77) on the VSS-A sub-scale. This indicates that vertigo/balance was a greater problem than autonomic/anxiety-related symptoms. Details of scores according to dizziness and diagnostic groups are presented in Table 3.

Simple logistic regression analyses showed that sex, symptom duration, neck pain, sway, and diagnosis, but not age, significantly predicted dizziness on the VSS-SF. Moreover, all the variables significantly predicted vertigo/balance-related symptoms, while sex, neck pain and sway predicted autonomic/anxiety-related symptoms. Diagnosis predicted significant reduced likelihood for development of symptoms when comparing non-otogenic dizziness to diagnoses in the vestibular category. The effect of diagnostic belonging with respect to vertigo/balance- and autonomic/anxiety-related symptoms varied. Details of the results are presented in Table 4.

**Table 4:** Unadjusted logistic regression

	**VSS-SF^a^**			**VSS-V^b^**			**VSS-A^c^**		
	
	**OR^d^**	**95% CI^e^**	**P^f^**	**OR^d^**	**95% CI^e^**	**P^f^**	**OR^d^**	**95% CI^e^**	**P^f^**
Sex (male/female)	1.59	(1.08, 2.34)	0.02	1.73	(1.17, 2.56)	0.01	1.53	(1.05, 2.23)	0.03
Age per 10 years interval	0.89	(0.75, 1.05)	0.17	0.78	(0.66, 0.94)	0.01	0.89	(0.75, 1.05)	0.16
Neck pain at medical examination	3.10	(1.84, 5.22)	< 0.01	2.66	(1.59, 4.45)	< 0.01	2.60	(1.62, 4.16)	< 0.01
Symptom duration (short ≤ 6 months)	2.46	(1.53, 3.95)	< 0.01	3.27	(2.02, 5.28)	< 0.01	1.39	(0.87, 2.20)	0.17
Sway per 1 m eyes closed	1.52	(1.10, 2.11)	0.01	1.65	(1.16, 2.35)	< 0.01	1.42	(1.05, 1.91)	0.02
**Diagnosis**			< 0.01			0.07			< 0.01
Non-otogenic dizziness	1.00	(ref.)		1.00	(ref.)		1.00	(ref.)	
Menière's disease	0.47	(0.26, 0.86)		0.60	(0.32, 1.01)		0.48	(0.27, 0.86)	
Vestibular schwannoma	0.29	(0.14, 0.61)		0.39	(0.18, 0.84)		0.31	(0.15, 0.64)	
Benign positional vertigo	0.52	(0.29, 0.97)		0.61	(0.33, 1.14)		0.54	(0.30, 0.95)	
Vestibular neuritis	0.54	(0.29, 1.00)		0.49	(0.27, 0.90)		0.64	(0.35, 1.15)	
Cervical dizziness	1.21	(0.56, 2.64)		0.99	(0.47, 2.08)		1.17	(0.57, 2.38)	

Unadjusted logistic regression analysis of dizziness according to the VSS-SF, vertigo/balance (VSS-V) and autonomic/anxiety-related symptoms (VSS-A) in N = 405 patients with complaints of dizziness examined in the period 1992–2001 in Bergen, Norway

^a ^VSS-SF: dichotomized at cut-off value ≤ 6.5, i.e. dizziness, yes/no

^b ^VSS-V: dichotomized at cut-off value ≤ 2.5, i.e. vertigo/balance-related dizziness, yes/no

^c ^VSS-A: dichotomized at cut-off value ≤ 3.5 points, i.e. autonomic/anxiety-related dizziness, yes/no;

^d ^OR: Unadjusted odds ratio

^e ^CI: Confidence interval

^f ^P-value from likelihood ratio test

The adjusted regression models left long-term symptom duration (> 6 months) and neck pain as significant predictors of dizziness. These two variables, in addition to sway and age, significantly predicted vertigo/balance-related symptoms, with age as a negative predictor. Neck pain was the only significant predictor of autonomic/anxiety-related symptoms (Table 5). Diagnosis was not a predictor in the adjusted analyses. Details of the results are presented in Table 5.

**Table 5:** Adjusted logistic regression

	**VSS-SF^a^**			**VSS-V^b^**			**VSS-A^c^**		
	
	**OR^d^**	**95% CI^e^**	**P^f^**	**OR^d^**	**95% CI^e^**	**P^f^**	**OR^d^**	**95% CI^e^**	**P^f^**
Sex (male/female)	1.28	(0.78, 2.09)	0.33	1.29	(0.77, 2.15)	0.34	1.44	(0.92, 2.26)	0.11
Age per 10 years interval	0.94	(0.76, 1.17)	0.60	0.76	(0.64, 0.96)	0.02	0.95	(0.78, 1.15)	0.58
Neck pain at medical examination	2.83	(1.47, 5.47)	< 0.01	3.22	(1.60, 6.48)	< 0.01	2.27	(1.30, 3.96)	< 0.01
Symptom duration (short ≤ 6 months)	2.86	(1.64, 4.97)	< 0.01	4.02	(2.27, 7.12)	< 0.01	1.41	(0.83, 2.39)	0.21
Sway per 1 m eyes closed	1.39	(0.94, 2.06)	0.80	1.58	(1.03, 2.42)	0.02	1.25	(0.90, 1.73)	0.17
Diagnosis			0.47			0.99			0.42
Non-otogenic dizziness	1.00	(ref.)		1.00	(ref.)		1.00	(ref.)	
Menière's disease	0.62	(0.30, 1.26)		0.96	(0.45, 2.05)		0.63	(0.33, 1.20)	
Vestibular schwannoma	0.39	(0.13, 1.16)		0.73	(0.22, 2.41)		0.41	(0.15, 1.17)	
Benign positional vertigo	0.69	(0.33, 1.43)		0.91	(0.42, 1.96)		0.64	(0.33, 1.24)	
Vestibular neuritis	0.87	(0.41, 1.84)		1.14	(0.52, 2.47)		0.88	(0.44, 1.74)	
Cervical dizziness	1.08	(0.43, 2.76)		0.97	(0.38, 2.47)		0.99	(0.83, 2.39)	

Multiple logistic regression analysis of dizziness (VSS-SF), balance (VSS-V) and autonomic/anxiety-related symptoms (VSS-A) in N = 405 patients with complaints of dizziness examined in the period 1992–2001 in Bergen, Norway

^a ^VSS-SF: dichotomized at cut-off value ≤ 6.5, i.e. dizziness, yes/no

^b ^VSS-V: dichotomized at cut-off value ≤ 2.5, i.e. balance-related dizziness, yes/no

^c ^VSS-A: dichotomized at cut-off value ≤ 3.5 points, i.e. autonomic/anxiety-related dizziness, yes/no

^d ^OR: Adjusted odds ratio

^e ^CI: Confidence interval

^f ^P-value from likelihood ratio test

There were no significant differences between responders and non-responders (n = 272) with respect to information of age, sex, sway parameters and symptom duration from debut to survey.

## Discussion

At the time of the survey, the whole sample had severe symptoms of dizziness years after onset, and dizziness was associated with vertigo/balance- as well as autonomic/anxiety-related symptoms in all diagnostic groups. Significantly more severe dizziness was seen in the non-otogenic compared to the vestibular schwannoma group. The majority (73%) confirmed current dizziness with recent episodes, and the mean (SD) symptom score in this group was 17.2 (10.1) on the VSS-SF scale, which defines severe dizziness ≥ 12 [15]. In the group claiming current dizziness, vertigo/balance was a greater problem than autonomic/anxiety. More than half of the patients reported neck pain at the time of the survey. Long-term symptom duration and neck pain identified in the medical examination, were the most prominent predictors. Sway and age had some impact on vertigo/balance-related problems. Diagnostic belonging was of some importance reducing the likelihood for developing symptoms in the vestibular category, but the effect disappeared in the adjusted analyses.

The severe and persisting dizziness was somewhat surprising, and the results should be interpreted with caution, as one third failed to return the questionnaire. It is possible that an attrition bias may have influenced the results negatively. It has been suggested that non-responders do not bother to reply because of fewer problems [17]. Responders and non-responders were similar with respect to background data. It has been suggested that early and late responders differ with respect to scores, and that late responders and non-responders have most similar scores [17]. In the current study the mean symptom scores between early and late responders did not differ on the VSS-SF and sub-scales (p range 0.69 – 0.90). It is possible that non-responders' lack of response have not influenced scores to any great extent. Forty-six forms were discarded because of too many missing items, and it is possible that these scores were similar to the non-responders'. Imputation of missing items was done in 40 forms. As maximum two items in each sub-scale were imputed, it is unlikely that this would have influenced the resultant scores to any great extent. The VSS-SF asks for self-reported symptoms within a relatively short period of time, but there is always a possibility of recall bias resulting in unrealistic negative (higher) *or *positive (lower) symptom scores [18]. It is difficult to assess these aspects, as dizziness is a highly subjective sensation, although it could be argued that self-reports represent the gold standard [1].

Symptom severity in the present study corresponded to findings in another study [19] with similar patients (n = 32) referred to our department (VSS-SF mean 15.8 SD 9.0). Studies in patients with persistent dizziness recruited from general practices in England, have shown symptom scores on the VSS-SF ranging from 10.9 to 16.6 [4,14,15]. The studies from England tended to include somewhat older patients (mean age around 60 years) and to have a greater percentage of women (70 – 80%) compared to the studies from our department, while symptom duration and diagnostic groups were similar [4,15]. Our findings were also similar to that seen in a group of slightly older patients with Menière's disease (17.3) recruited from a specialist centre [20].

In the present study vertigo/balance-related symptoms were a greater problem than autonomic/anxiety-related symptoms in line with findings in the other study from our department [19]. On the other hand Godemann and co-workers [21] reported higher autonomic/anxiety (VSS-A: mean 0.76, SD 0.77) than vertigo/balance (VSS-V: mean 0.33, SD 0.22) scores in patients following vestibular neuritis. This difference could possibly be explained by the frightening aspect of recent (6 months earlier) acute attacks in the study by Godemann and co-worker [21] as opposed to our groups' long-lasting problems. Two other studies [14,22] did not find any dominance with respect to the type of problems. Identification of symptom characteristics is important in relation to rehabilitation.

The majority of patients had vestibular disorders. The conditions are considered to be benign, and most, with some exceptions, have a short and self-limiting course [1,23]. In Menière's disease, symptoms have been reported even after 20 years [24], and the fluctuating nature of the condition may have influenced the scores. The severe dizziness seen in the vestibular schwannoma group was more surprising. These tumours are thought to lead to auditory rather than dizziness symptoms, but a recent study [25] showed that dizziness had the greatest impact on health-related quality of life. The natural history of benign positional paroxysmal vertigo indicates resolution [11], but recurrence is common and persistent dizziness over time has been reported [26] as well as balance problems [27]. In patients with vestibular neuritis, recovery from acute vertigo is within days/weeks [1]. However, residual balance problems are not unusual [11], and vertigo has been reported up to 8 years after the initial attack [28]. In the non-vestibular category (non-otogenic and cervical dizziness), symptom severity was similar to that of the vestibular category, but comparison with other studies are difficult due to lack of consistent classifications.

One explanation of the severity of symptoms could be related to patient selection, since a large proportion of patients were referred due to persistent symptoms, leading to more secondary health problems and possibly worse prognosis. Symptom severity might also be explained by the presence of co-morbid conditions at the time of the survey, although no association between dizziness and other chronic conditions were found. Physical inactivity over the years could also explain symptom severity. Early exercise as a means to enhance compensation has been documented [29], and the importance of being physically active was pointed out to all patients during the medical consultation. However, verbal information alone at this stage would most likely have been insufficient. For many patients, activity is associated with provocation of dizziness, and therefore avoided. In a study from Sweden on patients with Menière's disease, avoidance of activities was reported by 75% [30].

Long-term symptom duration and neck pain were the most important predictors of symptom development. Short-term duration of symptoms at referral (< 6 months) combined with programs of exercises is suggested to influence the prognosis positively [31]. A positive effect of vestibular rehabilitation has been reported from primary care [15]. The majority of patients are probably handled adequately in primary care [7], but for some, a closer follow-up might be required. For patients in the present study, there was no offer of organised vestibular rehabilitation at the time, and the general knowledge of early activity to promote compensation, may be questioned. Even when available, it is probably only a small percentage of patients that are referred to vestibular rehabilitation. In two studies from England referral rate to physiotherapy from general practice was reported as 2–6% [3,9]. However, in another study clustering patients based on presentation and impact of dizziness on function, 9%, 30% and 17% were referred respectively [6]. Lowest referral rate was seen among those with "non specific" dizziness with the most severe impact on function. In a study from Sweden [32], referral to physiotherapy was compared before and after an educational program. The program, which emphasised the active approach in vestibular rehabilitation, was directed at primary health care staff. The program had no influence on referral rate (p = 0.34), which remained around 10% [32].

As for neck pain, head instability associated with vestibular disorders [33] may result in dysfunctional head-on-trunk control strategies [34] putting excessive strain on the neck muscles over time. The combined effect of neck pain and dysfunctional control strategies may sustain balance problems [35], and in part explain the association between neck pain and balance. Neck pain was also found to be a predictor of autonomic/anxiety-related dizziness, possibly associated with a sensation of lack of control [36].

Our study showed that increased sway, i.e. physical signs of balance problems, had some predictive effect on vertigo/balance-related symptoms. Balance is negatively influenced by age [37], but in our study an increase in age was associated with less balance problems. This could be explained by adaptation to more sedate lifestyles as a result of dizziness. Badke and co-workers [38] reported younger age as a predictor of balance problem following surgical procedures for vestibular disorders, possibly because younger persons have expectations of more active lifestyles even when bothered with dizziness.

Being classified in the non-otogenic category increased the likelihood of developing problems compared to patients in the vestibular category. The first category rules out specific causes of dizziness, which in itself could result in sustained problems for psychological as well as physical reasons; i.e. fear of moving to avoid provocation of dizziness. However, in the adjusted analyses, there was no effect of diagnosis, which is in conformity with two other studies [5,36]. The most important predictor was perception of severity in the initial attack [36]. In another study poor prognosis was associated with vertigo, psychiatric etiology and disequilibrium [1]. It is possible that some of our patients, at the time of the medical examination, met the criteria for chronic subjective dizziness according to Staab and Ruckenstein, i.e. persistent, non-specific dizziness [39]. The condition cannot be explained by any active medical conditions [39], and thus unrelated to specific diagnosis. In some cases, an acute neuro-otologic condition may have started a process triggering anxiety, which in turn can predict chronic dizziness [39]. This has been suggested in patients with vestibular neuritis [21,39] and benign paroxysmal positional vertigo [39].

Classification of patients with long-lasting dizziness is not straight forward, and strict criteria could not always be applied, due to missing information in patient history data [40] and unclear diagnostic criteria [41,42]. While these factors represent limitations of the study, the diagnostic procedures probably reflect clinical reality in most otolaryngology departments better than in carefully designed prospective studies. In conjunction with the medical examination, the use of questionnaires could improve classification and identification of symptoms.

## Conclusion

In conclusion, this study shows that a large group of patients have persistent and severe dizziness influenced by vertigo/balance- and autonomic/anxiety-related symptoms years after the initial diagnosis. The wait-and-see attitude is problematic, as early and active exercises paying attention to the presence of neck pain is essential to promote compensation. Diagnoses have limited prognostic value. It is suggested that questionnaire-based evaluations could assist in classification of patients and identification of symptoms, and thereby provide a better basis for specific rehabilitation.

## Abbreviations

VSS: Vertigo symptom scale; VSS-SF: Vertigo Symptom Scale – Short Form; VSS-V: vertigo-balance sub-scale; VSS-A: autonomic-anxiety sub-scale; IQR: interquartile range; SD: standard deviation.

## Competing interests

The authors declare that they have no competing interests.

## Authors' contributions

KW designed the studies, collected data, performed statistical analysis and drafted the manuscript. AEL, FG, and SHGN all participated in designing the study and contributed in drafting the manuscript. GEE has participated in the statistical analysis and in drafting the manuscript pertaining to statistics and results. All authors have read and approved the final manuscript.

## Pre-publication history

The pre-publication history for this paper can be accessed here:


